# Tamdy Virus in *Ixodid* Ticks Infesting Bactrian Camels, Xinjiang, China, 2018

**DOI:** 10.3201/eid2511.190512

**Published:** 2019-11

**Authors:** Hong Zhou, Zhenghai Ma, Tao Hu, Yuhai Bi, Amutikari Mamuti, Runyuan Yu, Michael J. Carr, Mang Shi, Juan Li, Kirill Sharshov, George F. Gao, Weifeng Shi

**Affiliations:** Shandong First Medical University & Shandong Academy of Medical Sciences, Taian, China (H. Zhou, T. Hu, R. Yu, J. Li, W. Shi);; Xinjiang University, Urumqi, China (Z. Ma, A. Mamuti);; Chinese Academy of Sciences, Beijing, China (Y. Bi, G.F. Gao);; University College Dublin, Dublin, Ireland (M.J. Carr);; Hokkaido University, Sapporo, Japan (M.J. Carr);; Sun Yat-sen University, Guangzhou, China (M. Shi);; The University of Sydney, Sydney, New South Wales, Australia (M. Shi); Research Institute of Experimental and Clinical Medicine, Novosibirsk, Russia (K. Sharshov);; Chinese Center for Disease Control and Prevention, Beijing (G.F. Gao)

**Keywords:** Tamdy virus, Ixodid ticks, Bunyavirales, Xinjiang, phylogenetic analysis, ticks, viruses, China, bactrian camels, vector-borne infections, zoonoses, arboviruses

## Abstract

We isolated Tamdy virus (TAMV; strain XJ01/TAMV/China/2018) from *Hyalomma asiaticum* ticks infesting Bactrian camels in Xinjiang, China, in 2018. The genome of the strain showed high nucleotide similarity with previously described TAMV strains from Asia. Our study highlights the potential threat of TAMV to public health in China.

Tamdy virus (TAMV) was first isolated from the tick species *Hyalomma asiaticum asiaticum* collected from sheep in the Tamdinsky district of the Bukhara region, Uzbekistan, in 1971 (*1*). Subsequently, large-scale surveillance of TAMV from Ixodidae ticks using newborn mice successfully isolated 47 TAMV strains from various tick species from Armenia, Kazakhstan, Kyrgyzstan, Turkmenistan, and Uzbekistan, highlighting both its widespread distribution and its ability to infect mammals (*2*). Recently, TAMV was identified in Turkey from *Hyalomma* spp. ticks collected from *Meriones tristrami* gerbils in the Middle East (*3*). Sequence comparison and phylogenetic analyses of full-length TAMV genomes from different subspecies of *H. asiaticum* ticks taxonomically classified it in the genus *Nairovirus*, family Bunyaviridae (*4*). TAMV was also detected in 1973 from a febrile patient in Kyrgyzstan (*5*).

In May 2018, fourteen ticks attached to 2-humped Bactrian camels (*Camelus bactrianus*) were collected from a camel farm in Xinjiang, China. We extracted total RNA of the ticks using the E.Z.N.A. Total RNA Kit (Omega Bio-tek, https://www.omegabiotek.com). We used a transcriptomics approach to investigate the viruses harbored by the ticks and used the BGI mRNA Library Preparation protocol according to MGIEasy mRNA Library Prep Kit (BGI, https://www.bgi.com) to construct the RNA sequencing libraries for each tick. We conducted paired-end (100-bp) sequencing of each RNA library on the BGISEQ-500RS platform (BGI). We obtained 493,090,699 raw reads and then adaptor and quality trimmed them with the Fastp program (*6*), resulting in a total of 492,344,756 clean reads. These reads were de novo assembled using Trinity (*7*) with default settings. We compared the assembled contigs using BLASTn (http://blast.ncbi.nlm.nih.gov/blast.cg) against the nucleotide database downloaded from GenBank, with an E-value cutoff set at 1 × 10^−5^.

We identified contigs annotated as the large (L), medium (M), and small (S) gene segments of TAMV (family Nairoviridae, genus *Orthonairovirus*) in 1 tick (pool 10). To confirm the assembled viral contigs, we mapped reads back to the full-length genome of the TAMV strain LEIV-1308Uz (GenBank accession nos. KP792726–8, corresponding to the L, M, and S gene segments) as reference with Bowtie2 (*8*) and inspected using Geneious version 11.1.5 (Biomatters, Ltd., https://www.geneious.com). After removing repetitive reads, we mapped 34,172 reads to the L gene segment (depth 252 + SD 120), 60,184 reads to the M gene segment (depth: 1186 ± 418), and 8,724 reads to the S gene segment (depth 392 + 138). The virus genome obtained comprised L segment (encoding the RNA-dependent RNA polymerase [RdRp]), 12,215 bp; M segment (encoding the glycoprotein precursor), 4,565 bp; and S segment (encoding the nucleocapsid), 2,005 bp.

We then cultured the grinding fluid supernatant corresponding to pool 10 in Vero cells in Dulbecco Modified Eagle medium. We observed apparent cytopathic effects, such as higher cell refractive index, cell shrinkage, size reduction, rounding, and shedding, in infected Vero cells at 11 days postinfection (Figure, panel A). The virus strain was named XJ01/TAMV/China/2018 (hereafter XJ01). After 2 passages, we performed further transcriptome sequencing of the first- and second-generation virus suspension from cell cultures. We assembled the complete genome sequences of XJ01 again, as described, and found that the TAMV genomes from cell cultures were identical to those from the original sample.

**Figure:**
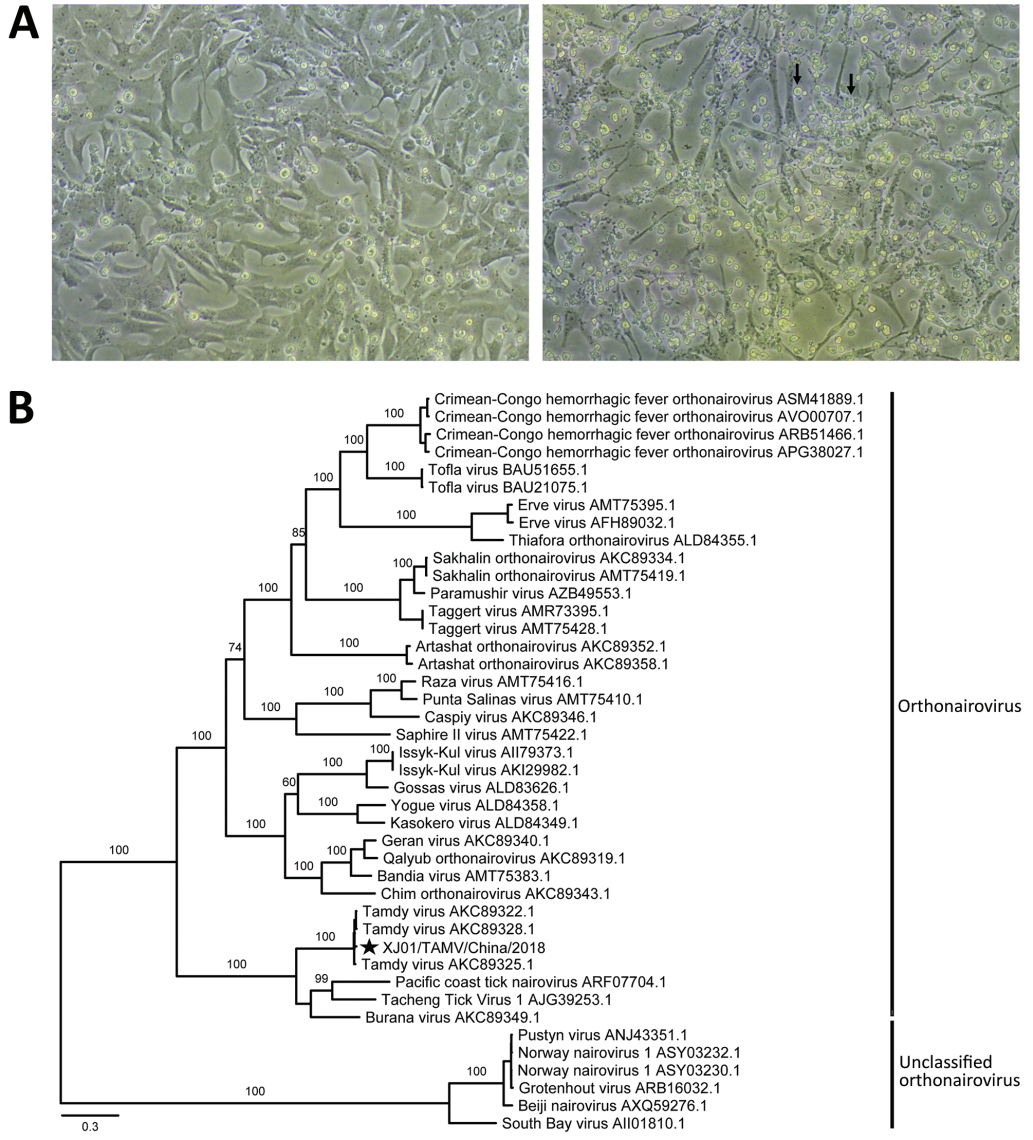
Identification of the Tamdy virus (TAMV) strain XJ01/TAMV/China/2018 from *Hyalomma asiaticum* ticks infesting Bactrian camels in Xinjiang, China, 2018, by cell culture and phylogenetic analysis. A) Light micrographs of cytopathic effects caused by TAMV infection at 11 days postinfection. Left, normal Vero cells as control; right, infected Vero cells with apparent cytopathic effects (black arrows). Original magnification ×100. B) Phylogenetic analysis of the RNA-dependent RNA polymerase protein sequences of TAMV and representative viruses in the family *Nairoviridae*. Scale bar indicates nucleotide substitutions per site. Star indicates strain from this study.

To confirm the genome sequence of XJ01, we designed 14 paired primers for Sanger sequencing (Appendix Table). The consensus gene sequences of Sanger sequencing of the amplified products were consistent with those from transcriptome sequencing and were deposited in GenBank (accession nos. MK757580–2). Sequence comparison revealed that XJ01 was highly similar to 3 previously described TAMV strains from Asia; sequence identities were 94.8%–94.9% for the L segment, 93.5%–94.7% for the M segment, and 95.4%–96.8% for the S segment.

Phylogenetic analysis of representative strains of the family *Nairoviridae* using RAxML (*9*) revealed that the 4 TAMV strains clustered with high bootstrap support and fell within the *Orthonairovirus* genus in the RdRp tree (Figure, panel B). In addition, they were closely related to several other orthonairoviruses from ticks, including Burana virus, Tacheng tick virus 1, and Pacific coast tick nairovirus in all the RdRp, glycoprotein precursor, and nucleocapsid trees and formed a small cluster in the *Orthonairovirus* genus (Figure, panel B; Appendix Figures 1, 2).

We also obtained the cytochrome c oxidase gene sequence of the tick and deposited it in GenBank (accession no. MK757583). A BLASTn search (https://blast.ncbi.nlm.nih.gov/Blast.cgi) revealed that the top hit was from *H. asiaticum* (GenBank accession no. KX882103.1) with sequence identity of 99%; this species is a widely distributed tick in China, especially in northwestern China (*10*).

In summary, we identified a TAMV strain from Ixodid ticks collected in Xinjiang, China, that poses a threat to public health in Xinjiang and even globally. Because of the ability of TAMV to infect mammals including humans, the lack of effective antiviral drugs and prophylactic vaccines, and the widespread distribution of its major host in China, extensive TAMV surveillance is urgently needed.

Appendix Additional information for study of Tamdy virus in Ixodid ticks infesting Bactrian camels, Xinjiang, China, 2018.
