# Supplementary material for: Tamdy Virus in Ixodid Ticks Infesting Bactrian Camels, Xinjiang, China, 2018
Source: Emerg Infect Dis. 2019 Nov;25(11):2136–8. doi: 10.3201/eid2511.190512 (PMC6810205; doi:10.3201/eid2511.190512)
Supplement: Appendix — Additional information for study of Tamdy virus in Ixodid ticks infesting Bactrian camels, Xinjiang, China, 2018. [file 19-0512-Techapp-s1.pdf]

Article DOI: https://doi.org/10.3201/eid2512.190512

Tamdy Virus in Ixodid Ticks Infesting Bactrian Camels, Xinjiang, China, 2018

Appendix

Appendix Table. Oligonucleotides used to amplify the TAMV XJ01 strain genome*

Primers (nucleotide positions)*	Sequence, 5′→3′	Product length, bp
Forward FL1 (1–23)	CACACCCAAAATACATAGAACCA	1,216
Reverse RL1 (1197–1216)	CGTCACGGACAGCTCCCAAA	
Forward FL2 (1110–1132)	TATTCACACACTTGCTACTCTCG	1,199
Reverse RL2 (2287–2308)	GCTTATATGCACTGGAAGAATC	
Forward FL3 (2184–2204)	TAAGCCCGATTTCTTGATAGC	1,190
Reverse RL3 (3353–3373)	CACTTCGTTTTGAGACCATGT	
Forward FL4 (3278–3298)	TCCTTGCATCCCAGATACAGC	1,302
Reverse RL4 (4557–4579)	TTCCTGATTGTTTACAGAAGTCG	
Forward FL5 (4509–4530)	AATAGGAATAAACAACGGTCAC	2,338
Reverse RL6 (6825–6846)	ACCTTCTGTCCTACCAGTCAAC	
Forward FL7 (6712–6731)	GCACTGAGCACCACAAGAGC	1,339
Reverse RL7 (8027–8050)	CACTATTATACATGGCTTGCTGAG	
Forward FL8 (7926–7945)	CCACCGTGTAACACCTGCTGTC	1,229
Reverse RL8 (9135–9154)	CGAGTGACCGGCCTGTGCTG	
Forward FL9 (9096–9116)	TGATCGATGGAATAGTAAGCA	3,120
Reverse RL10 (12196–12215)	CACACCCAAATACCATAAACAC	
Forward FM1 (1–22)	CACACCAAGCATTATAAACCAA	1,022
Reverse RM1 (1001–1022)	AGCTCAATGGTTAACAGCTCT	
Forward FM2 (940–959)	AAAGTCTGCAGCATAAGGGA	1,074
Reverse RM2 (1994–2013)	AACAACCAGGCCTAGCATCC	
Forward FM3 (1883–2004)	CAAAGCTGTCGGAGGATGAAGA	1,420
Reverse RM3 (3279–3302)	TCTGTGTGGAAGTACCAGTTCACC	
Forward FM4 (3175–3195)	TACTATACCTGCCATCCTGGG	1,391
Reverse RM4 (4544–4565)	CACACCAAGAAATCTTATCCAC	
Forward FS1 (1–24)	CACACCCAAACTTTACACTTTAGG	1,072
Reverse RS1 (1052–1072)	TCAACTTCCTTGGGCAATCCT	
Forward FS2 (950–972)	CCTTGTTCTAGCTCTCAATGACC	1,056
Reverse RS2 (1885–2005)	CACACCCAAAGCAAATTGCTC	

Values in parentheses indicate positions of the primer corresponding to the large, medium, small fragment of our Tamdy virus.

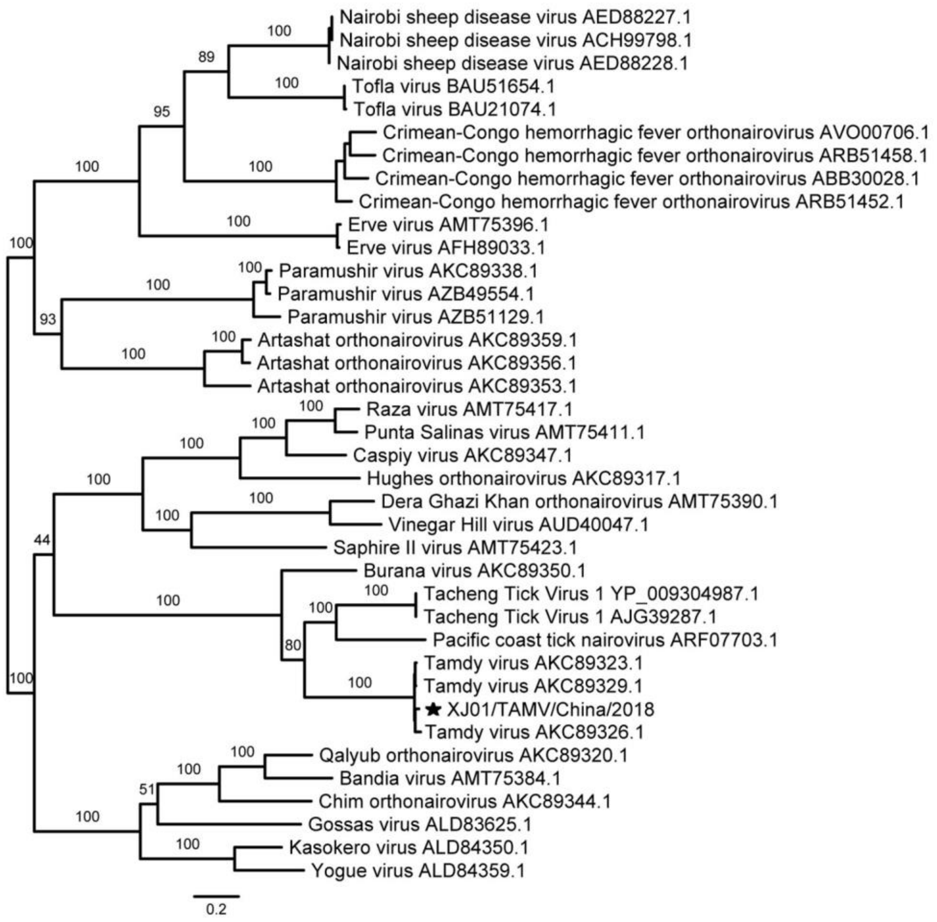

Appendix Figure 1. Phylogenetic analysis of the glycoprotein precursor sequences of TAMV and representative viruses in the family *Nairoviridae*. The Chinese TAMV strain in the present study is highlighted with a black star.

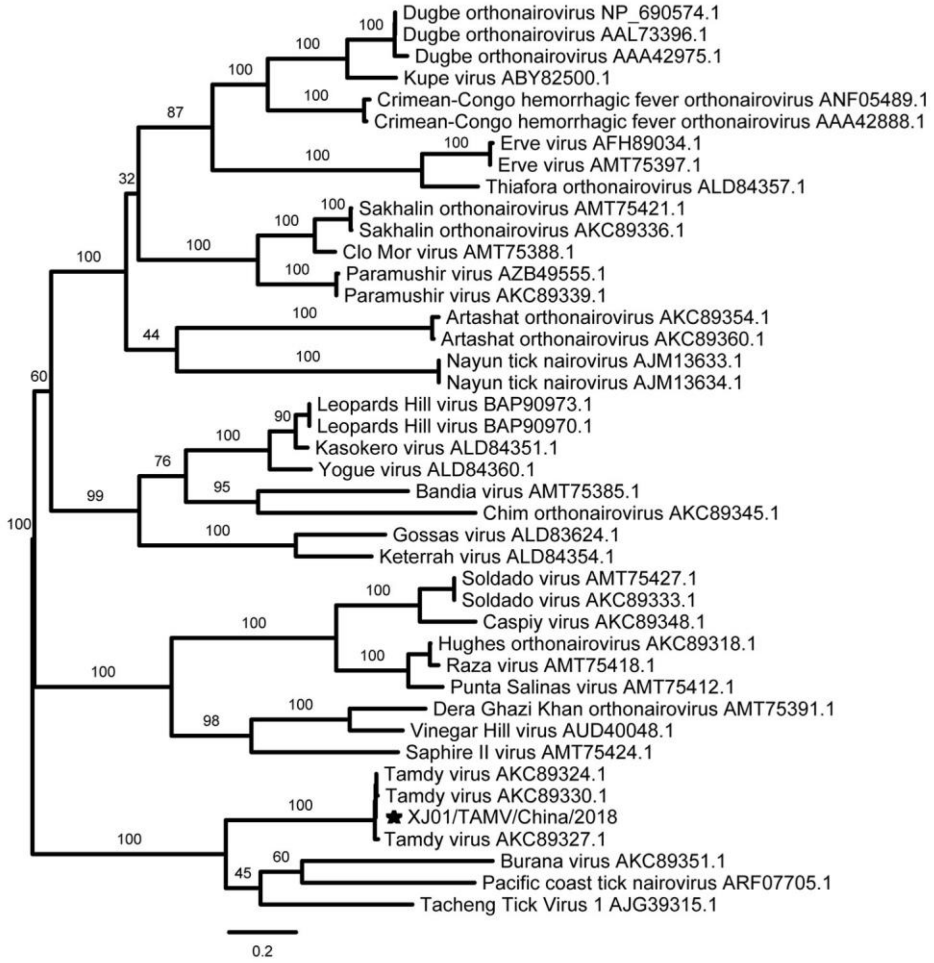

Appendix Figure 2. Phylogenetic analysis of the nucleocapsid protein sequences of TAMV and representative viruses in the family *Nairoviridae*. The Chinese TAMV strain in the present study is highlighted with a black star.
